# An Ecological Momentary Intervention to Reduce Alcohol Consumption in Young Adults Delivered During Drinking Events: Protocol for a Pilot Randomized Controlled Trial

**DOI:** 10.2196/resprot.6760

**Published:** 2017-05-25

**Authors:** Cassandra JC Wright, Paul M Dietze, Paul A Agius, Emmanuel Kuntsche, Robin Room, Michael Livingston, Margaret Hellard, Megan SC Lim

**Affiliations:** ^1^ Centre for Population Health Burnet Institute Melbourne Australia; ^2^ School of Public Health and Preventive Medicine Monash University Melbourne Australia; ^3^ Judith Lumley Centre La Trobe University Melbourne Australia; ^4^ Addiction Switzerland Lausanne Switzerland; ^5^ Behavioural Science Institute Radboud University Nijmegen Netherlands; ^6^ Faculty of Education and Psychology Eötvös Loránd University Budapest Hungary; ^7^ Centre for Alcohol Policy Research La Trobe University Melbourne Australia; ^8^ Centre for Social Research on Alcohol and Drugs Stockholm University Stockholm Sweden; ^9^ Department of Clinical Neurosciences Karolinska Institutet Stockholm Sweden; ^10^ Melbourne School of Population and Global Health University of Melbourne Parkville Australia

**Keywords:** alcohol drinking, young adult, mHealth, text messaging, ecological momentary intervention

## Abstract

**Background:**

Risky drinking is a significant public health issue in young Australian adults. Brief interventions are one of few effective methods of reducing risky drinking but are time and cost intensive; innovative methods of delivery are therefore of interest. Mobile phones offer new opportunities to collect data and intervene during risky drinking events. Mobile phones have successfully been used for delivery of alcohol-related brief interventions and data collection but not in combination with or during drinking events.

**Objective:**

This pilot study will investigate the efficacy of an ecological momentary intervention (EMI), with combined ecological momentary assessment (EMA) and brief intervention delivered by mobile phones to young adults during risky drinking events.

**Methods:**

We will use a 3-armed randomized controlled trial to investigate the efficacy of the intervention for reducing peak single occasion drinking. Our sample is recruited from an observational cohort study of young, risky drinkers. Participants will be randomized into 1 of 3 intervention arms. On 6 nights across a 12-week study period, EMI and EMA groups will complete hourly EMA surveys on their mobile phone. EMI participants will receive tailored feedback short message service (SMS) texts corresponding to their EMA survey responses. The EMI participants will not receive feedback SMS. A third group will have no contact (no-contact control). All groups will then be contacted for a follow-up interview within 4 weeks of the 12-week study period ending.

**Results:**

The primary outcome is mean reduction in standard drinks consumed during their most recent heavy drinking occasion as measured at follow-up. Secondary outcomes include alcohol consumption over the previous 6 months, experiences of alcohol-related harms, attitudes toward drinking and drunkenness, hazardous drinking and use of tobacco and illicit drugs. A random effects mixed modelling approach using maximum likelihood estimation will be used to provide estimates of differences in mean drinking levels between those receiving the intervention and control participants.

**Conclusions:**

This study is novel in that, unlike previous work, it will intervene repeatedly during single occasion drinking events. Further, it extends previous research in this area, which has applied limited tailoring of message content for SMS-based brief interventions. The findings of this study will contribute to the growing body of evidence to inform the use of mobile health interventions for reducing alcohol consumption and harms.

**Trial Registration:**

Australian New Zealand Clinical Trials ACTRN12616001323415; https://www.anzctr.org.au/ Trial/Registration/TrialReview.aspx?id=369534 (Archived by WebCite at http://www.webcitation.org/ 6qDqBZV9b)

## Introduction

Risky single occasion drinking (RSOD, sometimes termed binge drinking) remains a significant public health issue in Australia for young people. The 2013 National Drug Strategy Household Survey reported that approximately two-thirds of 20- to 29-year-olds engaged in RSOD in the past year (defined as 5 or more Australian standard drinks, or 50 g alcohol in a session) [1]. In 15- to 24-year-olds, 1 in 5 hospitalizations and 1 in 7 deaths are attributable to alcohol consumption [2]. Other associated harms include physical and sexual violence, risky sexual behavior, and both short- and long-term brain impairment and cognitive deficits [3-5].

Few individually targeted strategies have been shown to effectively change young people’s drinking behaviors. A notable exception is the brief intervention, which typically involves screening and assessing drinking behavior and providing tailored, personalized feedback. Although traditionally delivered in clinical settings, brief interventions have been successfully trialed in college and university settings and have shown efficacy in reducing alcohol consumption [6-9]. Brief interventions are traditionally delivered in face-to-face mode, which means that while effective, they are both time and resource intensive. Recent efforts have attempted to upscale this approach using Web-based and mobile phone delivery of brief interventions [10-16], with some success. Building on some of the works by Kypri [10,17], Voogt et al [14] developed the “What Do You Drink” Web-based brief intervention, combining weekly Internet-based screening with tailored feedback. The study found a significant reduction in weekly alcohol consumption in the experimental arm compared to the control arm at 4 points of follow-up. Newer technologies can provide opportunities to expand on this concept further and explore in-event characteristics, behavior, and intervention.

Approximately 95% of young adults in Australia own smartphones with computerized functions and Internet connections [18]. The integration of mobile phone use into young people’s lives provides new opportunities for health interventions to be delivered directly to at-risk populations during risky events such as drinking occasions.

Mobile phones have also been used as remote data collection tools. This data collection functionality means that they can serve as a platform for the screening and assessment phase of brief interventions, using ecological momentary assessments (EMAs) [19], which are repeated, real-time behavioral surveys. Previous studies have demonstrated the feasibility of collecting EMA data on specific occasions of alcohol use by young people using mobile phones [20]. Kuntsche and Labhart developed the Internet-based cell phone–optimized assessment technique (ICAT) [21] to collect alcohol consumption data from more than 200 Swiss young adults, using repeated EMAs between 5 PM and 11AM on weekend nights. They found this method to be easy and convenient and were able to record more than 10,000 questionnaires across the 5-week study period [20].

Recent studies have attempted to combine EMA with brief intervention (sometimes called ecological momentary intervention [EMI] or screening with brief intervention [SBI]). Suffoletto et al [16] used short message service (SMS) data collection with young adults reporting their intentions to drink on the coming weekend, commitment to reduce drinking, and later, their actual weekly drinking. Tailored advice was then sent to participants in response; they reported a small reduction in binge drinking within the sample of young adults. Riordan et al [12] collected alcohol consumption data and delivered brief advice by SMS message to university students in New Zealand. In that study, participants were sent 4 total EMA during orientation week (the week prior to the university semester starting, which usually involves multiple parties and other social events) and once per week during the semester. Participants in the EMA-EMI condition additionally received SMS with health consequence warnings at 7:30 PM on each night of orientation week. They found a reduction in alcohol consumption during orientation week for women but not for men [12].

However, we could find no studies which combined repeated alcohol consumption data collection with repeated SMS brief interventions delivered during single occasion drinking events. EMA captures subjects’ behaviors and experiences in real time, maximizing ecological validity by assessing subjects in their natural environment [22]. Another strength of the EMA is that it can reduce recall bias (retrospective reporting is known to underreport consumption). EMI offers both of these benefits with the added advantage of intervening in a participant’s natural environment while the targeted behavior is occurring.

This study uses an EMI, combining mobile Web-based EMAs (ie, surveys completed in a mobile phone Web-browser, similar to Kuntsche and Labhart’s ICAT) [21] and SMS brief interventions delivered during drinking events using mobile phones. The intervention was codesigned and evaluated by young people in a development study [23]. In the development study, participants deemed the intervention content, mode of delivery, and level of burden to be highly acceptable and feasible [23]. This research demonstrated both feasibility and acceptability; the next phase will determine whether this intervention is effective in changing behavior. This study aims to determine the impact of a tailored brief intervention delivered by mobile phone on young people's RSOD behavior.

## Methods

### Study Design

**Figure 1:**
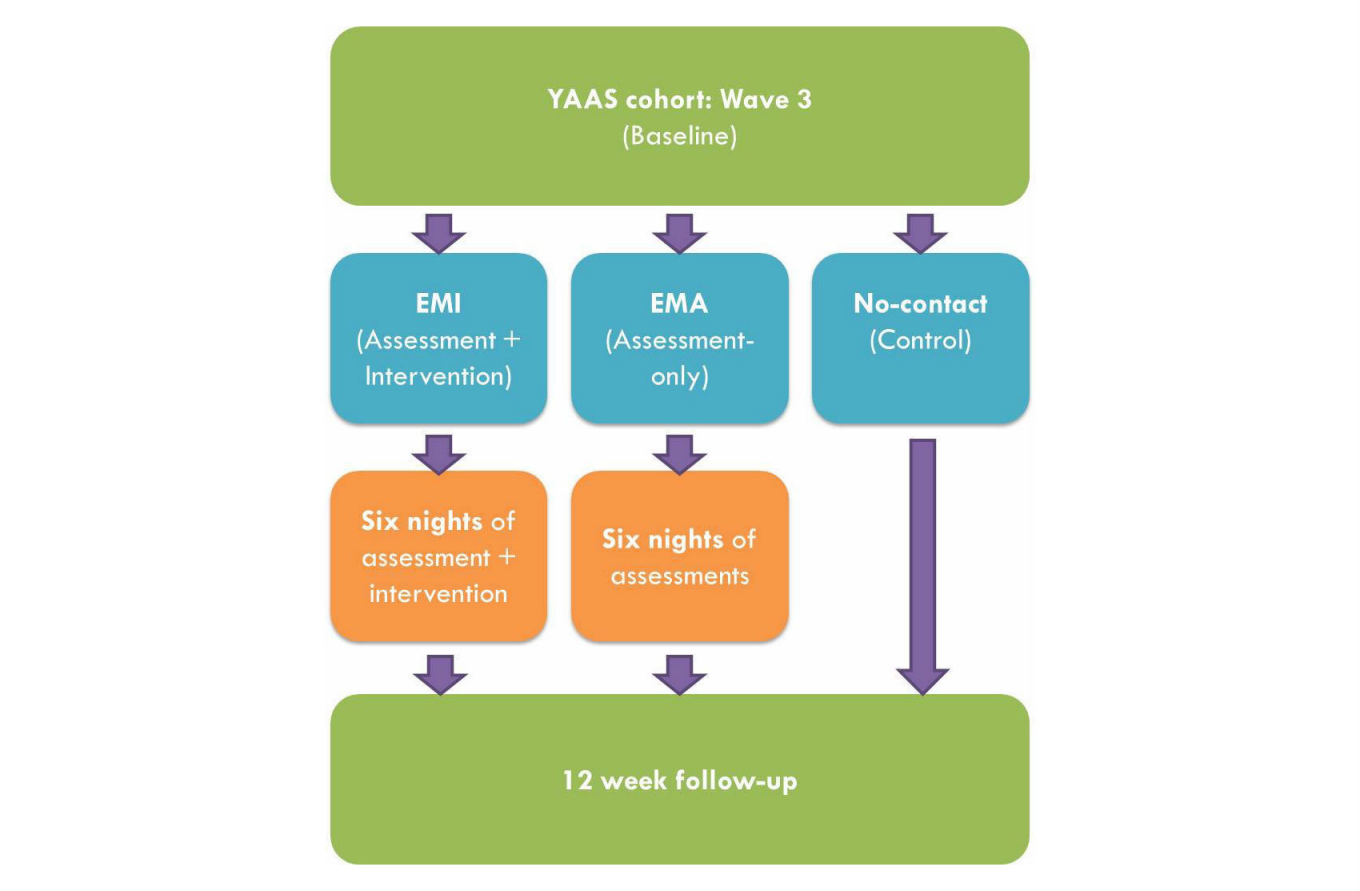
Study design.

We will use a 3-armed repeated measures randomized controlled trial (RCT) design to generate high-quality evidence of the impact of our mobile phone intervention on peak consumption of alcohol in risky drinking events among young people. The design of the study is summarized in Figure 1.

### Participants

Participants will be recruited from the ongoing Young Adults Alcohol Study (YAAS) cohort [24]. The YAAS cohort study commenced in 2012 and originally recruited a representative sample of 802 young Melburnians aged 18 to 25 years, screened for engagement in very high-risk drinking [24]. YAAS participants were recruited through random digit dialing from a sampling frame of landline telephone numbers. Eligible participants were administered a structured questionnaire using computer-assisted telephone interviews (CATIs) that included questions on demographics, past-year alcohol consumption, the Alcohol Use Disorders Identification Test (AUDIT) [25], drinking consequences and contexts [26,27], life satisfaction [28], and the most recent heavy drinking occasion [24], focusing on timing, locations, amount and types of alcohol consumed, and amount spent on alcohol at each location. Approximately half of the original sample were male (407/802, 50.7%), studying full-time (407/800, 50.9%), educated to a year 12 level (416/799, 52.1%), and had more than Aus $160 (US $118) weekly recreational spending money (416/788, 46.7%). Most were born in Australia (722/802, 90.0%), lived with parents (736/802, 91.8%), and identified as heterosexual (738/800, 92.3%) [24].

A second wave of data collection was completed in 2013 and a third in 2015; data from the final wave will be used as the baseline for this study. In 2013, 531/802 participants (66.2%) remained from the original cohort. In 2015, a total of 373/802 (46.5%) participants remained. Participants have not previously experienced any specific interventions as part of this study other than these telephone surveys.

### Recruitment and Screening

All participants who meet eligibility criteria will be invited to receive information regarding a new study. Eligibility criteria include owning a smartphone and recent risky drinking behavior (5 or more drinks in a single session in the past 3 months).

Those who agree to receive information will be randomly allocated to 1 of 3 arms: an intervention group where participants receive a brief intervention delivered over mobile phone (EMI) or to 1 of 2 control groups where participants will either complete EMAs without brief intervention (EMA) or do not receive any contact through the trial period (no-contact control). Participants will only receive study procedure information relating to the arm to which they have been allocated. The nature of the intervention means it will not be possible to blind participants; however, they will remain unaware of the detailed procedures of the other arms. Research personnel administering the follow-up questionnaires will be blind to the group to which participants were allocated.

Following group allocation, participants will be sent an SMS message containing a brief description of their arm of the study with a link to the participant information and consent form. Participants who do not respond will be sent 1 reminder SMS message 2 weeks later. Remaining nonrespondents will then be followed up by phone, up to a maximum of 6 phone calls each or until contact is made.

### Description of the Ecological Momentary Assessment Data Collection

Each participant from the EMI and EMA groups will be expected to choose 6 nights to complete EMA throughout the 12-week study period. We chose this number of events based on recommendations from the development study. No minimum criteria will be given to participants for amount of consumption planned; they will simply be asked to choose nights on which they are planning to drink. Participants will be provided with initial instructions on how to register for a night and will be prompted each Thursday night with a reminder to register if there are any nights over the weekend that they plan to drink. This process includes them sending a simple SMS message saying “Drink” on the day or they can register in advance by texting the name of the day of the week they intend to complete the intervention on (eg, “Saturday” for the upcoming Saturday). Participants will receive a confirmation message once they have registered.

The timing and frequency of the data collection and intervention design were informed by previous research in Switzerland [19] and Australia [23] which found that hourly surveys were perceived to have low burden. At 6 PM on chosen intervention nights, participants will receive a short SMS message asking them to complete their first survey with a link to an online questionnaire hosted by the online survey tool SurveyGizmo [29]. SurveyGizmo was chosen above other options due to mobile compatibility, visual appeal, and range of question types. The 6 PM presurvey will ask questions relating to their intentions for the night, including how much they plan to drink, spend, and eat; a ranked list of particular adverse events they wish to avoid (eg, vomiting, not being able to get home); their planned mode of transport home; next day plans; any alcohol consumption so far; mood; and the option of writing a message to themselves to be sent back to them during the night.

At hourly intervals between 7 PM and 2 AM, respondents will be sent a shorter EMA survey that asks about current location type, alcohol consumption since last survey, spending, mood, and perceived drunkenness. Participants will also be able to opt out of the intervention at the end of each survey if they are finishing their evening.

**Figure 2:**
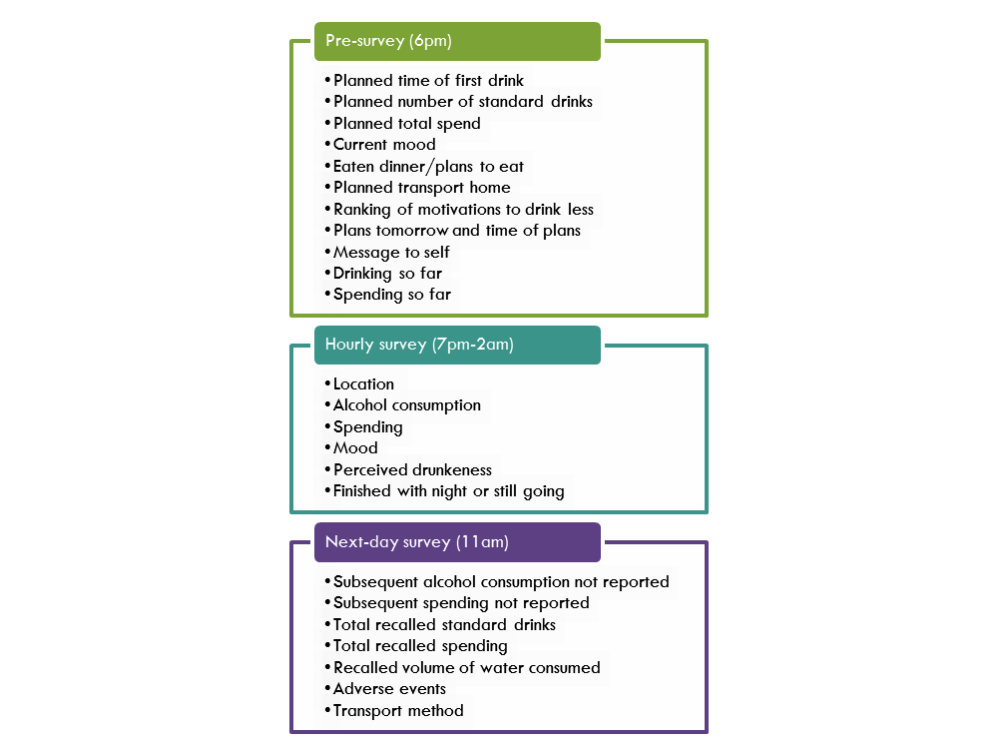
Data collected during drinking event.

At 11 AM the next day, participants will be sent another SMS with a mobile survey link. This survey asks for any alcohol consumption and spending occurring after the final EMA, an estimated total standard drinks and spending for the night, an estimated volume of water consumed for the night, reporting of adverse events, hangover experienced, and a fun rating of the night. Figure 2 summarizes the different data that will be collected during the drinking event at different time points.

### Intervention Group

In addition to completing EMAs, the EMI group will receive the SMS brief intervention component. Following submission of each EMA survey throughout intervention nights, they will receive a feedback SMS comprising of motivational information tailored based on their responses.

**Figure 3:**
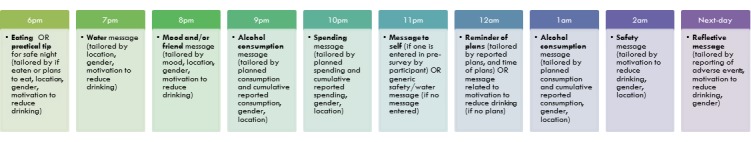
Framework for determining message tailoring.

All feedback SMS during the night will be comprised of information aiming to remind the EMI group participant of their original intentions or motivations, tips to avoid adverse consequences, or feedback relating to cumulative consumption or spending. These messages will be based on a different key variable each hour (see Figure 3).

This intervention, including all questionnaires, message content, and overall study features, was codesigned with a group of 42 young people in a development study conducted in 2014 [23]. This study involved 3-hour workshops to inform the design, individual testing of the intervention on a night of drinking, and evaluation involving both in-depth interviews and a structured online survey.

### Message Tailoring

A bank of messages has been created with over 15,000 messages contained within a matrix of key variables related to information collected about and from participants during the EMAs. Messages have been tailored to correspond with different situational contexts that participants may find themselves in throughout their drinking events. Messages were written and categorized according to gender as well as variables collected during the surveys such as varying locational contexts (ie, for a participant at a bar, as opposed to drinking at home), times of night, mood, motivations reported by participants, combinations of planned drinking versus actual drinking, and planned versus actual spending. The content, language, and framing of content was informed by the participatory codesign process used in the development study [23] with messages refined according to principles of motivational interviewing theory [30].

In order to create a feasible tailoring system, we created a framework to determine the type or topic of message sent at each hourly interval, with decision logic based on different variables collected throughout the night. Our SMS system was custom-developed by SurveySignal [31] according to our specifications. The SurveySignal system was designed so that once a participant triggered their drinking event by SMS (as described earlier), the system would generate a schedule of SMS with survey links to be sent hourly to that participant for the chosen night. If a participant opted out during the course of the night, the remaining schedule would be deleted so that no further surveys would be sent on that occasion. The SurveySignal system linked with SurveyGizmo (which we used to host our EMA surveys) using http POST so that data for the variables used in tailoring would be transmitted immediately to the SurveySignal database as soon as entered into the survey. The back end of SurveySignal has a databank of messages that were individually written to correspond to different combinations of the tailoring variables. These combinations are described in Figure 3. For each survey filled out, algorithms are run within SurveySignal to match an individual’s responses against the logic framework to determine which SMS should be sent. The system then generates and sends the matching SMS immediately to the participant. For example, at 6 PM, if the participant has indicated that they have not eaten and have not made plans to eat, they will receive a message about why it is important to eat with the particular message tailored by what they have input as something they would like to avoid that night (as a proxy for motivation to reduce drinking). A participant who reports in their presurvey that they would like to avoid feeling sick or vomiting may receive a message such as “Best way to not get sick tonight is to make sure you eat enough ASAP, and start with a big glass of water. Get on it!” In testing, most feedback SMS were received within 15 seconds of the survey completion.

### Control Groups

The first control group (EMA) will follow the EMA data collection procedure described above (including registration for 6 intervention nights and all surveys on each night) but will not receive any feedback SMS. This EMA-only group is necessary to investigate reactivity and the potential that—although unlikely [32]—completing assessments alone (without any feedback or other intervention) can affect drinking behavior. A second control group (no-contact control) will not receive any contact until follow-up, which will occur 12 to 16 weeks after the baseline assessment. The no-contact control group will be the primary control group used for comparison to the EMI group in analysis.

### Reimbursement

Participants from the EMI and EMA groups will receive reimbursements varied based on the level of participation in the study. For each event completed (up to a total of 6), participants will receive Aus $10 (US $7). If all 6 are completed, a bonus Aus $20 (US $15) is applied. Participation in the follow-up survey is valued at Aus $20 (US $15). Therefore, participants who complete all 6 events and the follow-up interview will receive Aus $100 (US $74) in cash or voucher. Participants from the no-contact control group will receive Aus $20 (US $15) for participating in the follow-up phone survey.

### Ethics

Ethics approval for the RCT has been obtained from the Monash University Human Research Ethics Committee. Ethics approval for the YAAS cohort study has been obtained from the Alfred Health Research Ethics Committee.

### Primary Outcome Measure

All measures were defined a priori. The primary outcome measure will be change in the mean peak number of drinks consumed in a single night over the 3-month intervention period between those receiving the intervention (EMI group) and control participants (no-contact control group). Our focus on heavy drinking from an occasional or binge perspective is because this is the main risky drinking pattern in our target age group [33,34]. We will assess this by asking participants about the number of drinks consumed in their last heavy drinking occasion in the past 3 months at both baseline and immediately following the intervention period. We expect to see a greater reduction in mean peak drinking in the EMI group compared to no-contact control group. In the first wave of YAAS [33], the mean number of drinks consumed on the most recent heavy drinking occasion was 13.1 (SD 5.2) Australian Standard Drinks. We estimate that reducing this by a mean of 2.5 standard drinks (*d*=0.48) would halve the odds of serious injury [35,36]; this forms the basis of our target effect size.

### Secondary Outcome Measures

Secondary outcomes of interest will also be collected at both baseline and follow-up interviews and include:

- Alcohol consumption (graduated quantity frequency)
- Alcohol-related harm (derived from the Gender, Alcohol, and Culture: An International Study [GenACIS] [27] and Victorian Youth Alcohol and Drug Survey questionnaires [37])
- Attitudes toward drinking and drunkenness (derived from GenACIS [27])
- Hazardous drinking (derived from World Health Organization AUDIT [25])
- Use of tobacco, illicit drugs, and nonmedical use of pharmaceuticals (derived from GenACIS [27])
- Life satisfaction (Personal Wellbeing Index [28])

Feasibility and acceptability will be assessed in the follow-up survey among both EMI and EMA groups using 5-point Likert scales asking participants to rate a series of individual statements pertaining to their experience of undertaking the intervention (eg, “The assessments were easy to complete” and “The feedback provided was relevant to me”). Additional process evaluation measures such as participant levels of response, refusal, and timeliness of response will be explored to assess feasibility and acceptability.

### Randomization

Using the Stata statistical software package version 13.1 (StataCorp LLC), block randomization will be conducted to ensure balanced randomization to each of the 3 study arms. Randomization will be completed by a researcher with no involvement in the trial.

### Sample Size

Study sample size is based on this primary aim to reduce mean peak consumption by 2.5 drinks in the intervention group (EMI) compared to controls (EMA and no-contact control). Assuming a standard deviation of peak drinking of 5.2, conservative 67% endpoint participation, 90% power, and 5% significance, it was estimated that a sample of 127 participants per group was required. The sample size estimate has been calculated to test for a group by time interaction from a mixed repeated measures design and a moderate correlation between subject measurements (*r*=0.45, estimated from wave 1 and 2 of YAAS cohort data).

### Statistical Analyses

Given the randomized design, dependency on repeated participant observations, and potential bias from study attrition, a random effects mixed modeling approach using maximum likelihood estimation will be used to provide unbiased estimates (assuming a missing at random missing data process) of differences in mean drinking levels between those receiving the intervention (EMI group) and control participants (no-contact control group). Study subjects and treatment will be modeled as random and fixed factors respectively in these mixed model analyses. The interaction between intervention and study time (baseline versus 12-week follow-up) is the primary focus of analysis. Distributional assumptions of models will be tested in the data and appropriate transformations applied where these are not reasonably met. These analyses will be repeated as secondary analyses to determine the impact of the assessments alone (ie, comparison of EMA and no-contact control groups) using participant observations from EMA assessment. Further analyses may also be undertaken to investigate associations between particular types of messages and alcohol consumption.

## Results

Enrollment for this study was completed in April 2016. Data analysis is currently being undertaken, and we expect to submit our results for publication in mid-2017.

## Discussion

### Summary

This study is novel in that, unlike previous work [12,15,16,20], it is designed to intervene repeatedly during single occasion drinking events. Further, it extends previous research in this area [12,38], which has applied limited tailoring of message content for SMS-based brief interventions. Participants in this trial have been screened multiple times for risky drinking behavior, and as such, are an ideal cohort to target with an intervention. A further strength of the study is the rich data collected through multiple sources (CATI interviews and EMA data).

### Limitations

A number of limitations also exist. While the intervention has been developed and tested through participatory methods, the development study [23] only tested the intervention on one night for each participant. Adherence may be affected by requiring multiple testing occasions.

Recruitment and adherence may also be affected by the nature of the sample. Participants have already completed multiple waves of data collection across 4 years, and it is possible that participants will be less inclined to participate in another, more extensive, study. The sample consenting to participate in the RCT may end up being comprised of those more motivated to participate in research or change their behavior. Analyses of nonrespondents and refusals will be undertaken in order to investigate this possibility.

The study relies on self-reported data, which has the potential for reporting bias; participants may either intentionally or unintentionally report inaccurate data through either EMAs or the telephone surveys. Participants will be given flexibility with choice of intervention nights and are not incentivized for actual consumption in any way to minimize the risks of either encouraging drinking or falsely reporting consumption. Further, a key challenge of collecting data during a drinking event is that participants will be consuming alcohol while reporting. This will not affect our assessment of the intervention’s efficacy because the primary outcome measure does not use EMA data. However, any future use of this data will be affected by this limitation, and we have implemented some measures to minimize this bias. For example, the surveys were designed to be as easy to complete as possible, in case of physical incoordination. Secondly, the surveys were hourly to give participants a shorter (and hence more easy to recall) reporting period. However, it is not entirely possible to eliminate this bias given the nature of in-event intervention. While validation with observational methods is beyond the scope of this study, data from EMAs during the night will be compared to next-day recall of alcohol consumption. There is also the possibility that intoxication may reduce the intervention message salience; however, this is yet to be tested. Another limitation of our approach is that complex tailored interventions do require more participant involvement than nontailored interventions. While our development study showed very high response rates (90% of all EMA surveys were completed), adherence in the current and other samples will not be known until tested. Finally, as with all incentivized intervention studies, adherence may not be able to be replicated outside of research settings.

### Conclusion

This study aims to investigate the efficacy of a mobile EMA and brief intervention for reducing risky drinking in young Australians. The findings of this study will contribute to the growing body of evidence [12,16,19] to inform the use of mobile health interventions for reducing alcohol consumption and harms.

## Abbreviations

AUDIT: Alcohol Use Disorders Identification Test
CATI: computer-assisted telephone interviews
EMA: ecological momentary assessment
EMI: ecological momentary intervention
GenACIS: Gender, Alcohol, and Culture: An International Study
ICAT: Internet-based cell phone–optimized assessment technique
NHMRC: National Health and Medical Research Council
RCT: randomized controlled trial
RSOD: risky single occasion drinking
SBI: screening with brief intervention
SMS: short message service
YAAS: Young Adults Alcohol Study
